# An Experimentally Based Micromechanical Framework Exploring Effects of Void Shape on Macromechanical Properties

**DOI:** 10.3390/ma15124361

**Published:** 2022-06-20

**Authors:** Sara Eliasson, Mathilda Karlsson Hagnell, Per Wennhage, Zuheir Barsoum

**Affiliations:** 1 Scania CV AB, SE-151 87 Södertälje, Sweden; 2 Centre for ECO^2^ Vehicle Design, SE-100 44 Stockholm, Sweden; wennhage@kth.se (P.W.); zuheir@kth.se (Z.B.); 3 Department of Engineering Mechanics, KTH Royal Institute of Technology, SE-100 44 Stockholm, Sweden; 4 Materials and Production, Polymers, Fibers and Composites, RISE Research Institutes of Sweden, SE-164 40 Kista, Sweden; mathilda.karlsson.hagnell@ri.se

**Keywords:** CFRP, porosity, multi-scale modeling, representative volume elements, microstructure

## Abstract

A micromechanical simulation approach in a Multi-Scale Modeling (MSM) framework with the ability to consider manufacturing defects is proposed. The study includes a case study where the framework is implemented exploring a cross-ply laminate. The proposed framework highlights the importance of correct input regarding micromechanical geometry and void characteristics. A Representative Volume Element (RVE) model is developed utilizing true micromechanical geometry extracted from micrographs. Voids, based on statistical experimental data, are implemented in the RVE model, and the effects on the fiber distribution and effective macromechanical properties are evaluated. The RVE algorithm is robust and maintains a good surrounding fiber distribution around the implemented void. The local void fraction, void size, and void shape affect the effective micromechanical properties, and it is important to consider the phenomena of the effective mechanical properties with regard to the overall void fraction of an RVE and the actual laminate. The proposed framework has a good prediction of the macromechanical properties and shows great potential to be used in an industrial implementation. For an industrial implementation, weak spots and critical areas for a laminate on a macro-level are found through combining local RVEs.

## 1. Introduction

The commercial vehicle industry’s pursuit for lightweight vehicles is intensifying with the advances of Battery Electric Vehicles (BEVs). Apart from reducing fuel consumption and CO_2_ emissions, a lighter structure leaves room for more batteries, increasing the driving range of BEVs [1]. Optimized lightweighting can be achieved through the introduction of Carbon Fiber Reinforced Plastics (CFRP). CFRP has a high strength-to-weight ratio and superior mechanical properties. However, due to its composition of different constituents, a CFRP material exhibits a more complex failure behavior compared to traditional metallic materials and needs to be described by several different failure and damage mechanisms simultaneously. For a CFRP component, the failure events at the micromechanical scale advance and initiate failure events at higher scales (meso- and macro-scale), preferably modeled using multi-scale models [2].

Multi-Scale Modeling (MSM) passes information between different length scales, and the interest in MSM methods has grown in the last few decades [3], especially in the automotive industry, where fast product development times and low-cost margins promote the need for simulation-driven design approaches. Saving time and cost, minimizing experimental techniques, the simulation methods used in a design process are indispensable. MSM is mature and highly valuable when implemented and could provide the extra information needed for a reliable and efficient detailed analysis of more complex materials, such as CFRP. The MSM methodology is successfully implemented in many areas of application, e.g., fiber- and braid-reinforced coil spring [4], wind turbine blades [5], and virtual testing of composites [6].

Simulations should be used as a driver to guide the designers early in the design process. More detailed and complex models would require increased computational time, and when introducing new materials and new methods, it is important to weigh between complexity and cost, cost in terms of time and money [7]. Yuan and Fish [8] introduced a framework to promote the use of computational homogenization in the industry. Okereke et al. [9] followed the trend by developing a virtual framework focusing on the prediction of the elastic properties of Unidirectional (UD) composites. In a recent study, Sun et al. [10] integrated an MSM method in a full framework, providing design guidance for failure events of woven and chopped Sheet Molding Compound (SMC) carbon fiber composites. A challenge with the MSM methods is to find an appropriate framework that supports the continuous use of the method. The requested framework needs to be broad and have the possibility to cover multiple areas, such as failure and fatigue. However, for the framework to be continuously used, there is a need for a good enough simulation approach that is accessible with the possibility for a more detailed analysis.

Adding to the complexity, component geometry, different manufacturing approaches, and different stages of automation give rise to different manufacturing defects, and producing a defect-free composite is highly expensive. For the modern manufacturing techniques that target lower production costs and time, it is inevitable to avoid defects and void formation [11]. The paradigm of Defect Damage Mechanics aims to quantify the production process to minimize cost, while still fulfilling the mechanical requirements [12]. Defects can have detrimental effects on the damage behavior of a composite, and attempting to capture the true micromechanical geometry and behavior, defect assessment will be essential for an industrial application.

Researchers have worked with the effects of voids, both experimentally and theoretically. Since voids are arbitrary and difficult to induce intentionally, statistical and numerical models are commonly implemented. Many studies simplify the true shape of voids [13,14,15], while other researchers highlight the importance of the void shape [16,17]. Statistical void data are essential to consider trying to capture the true behavior of the material modeled [18]. Most studies have a numerical approach when analyzing the effects of voids. However, more work considering actual experimental data is needed to understand what is important and how to apply it in an industrial simulation framework.

In order to design for life, efficient computational methods that can evaluate failure envelopes considering the choice of manufacture are needed. For commercial vehicles, the design lead times are decreasing, and there is a great need for early-stage models that can quickly assess the failure behavior of a composite structure and find weak spots and critical areas. To meet the needs of the expanding design guidelines of the commercial vehicle industry, a new modeling framework is presented, referred to as EMMMA; an Experimental Micromechanical Multi-scale Approach. Within the research frame on MSM methods for CFRP applications, it is important to develop a simplified approach with qualitatively good accuracy to be industrially applicable. This motivates the work reported within this study. The MSM framework focuses on the micromechanics utilizing Representative Volume Elements (RVEs) based on experimental statistical data to extract effective macromechanical properties. The framework is built on a modular approach where the material system and manufacturing method can be exchanged by exchanging the experimental input data with new experimental data from other material systems.

The MSM framework meets the need for a framework that employs the possibility to also model defects, i.e., voids. The study investigates the interaction of void shape, size, fraction, and distribution connected to an RVE model and a composite laminate. The study employs the concept of the RVE to bring results from a detailed microscale model into an efficient engineering macro-level approach, bridging the gap between manufacturability and microscopic simulations for an industrial implementation. The modeling of voids is based on experimental data and the local void fraction. Identifying weak spots and critical areas in a macro-model would be conducted by using a combination of RVEs varying the local void fraction. In this study, the material system used is a UD CFRP prepreg manufactured with heat compression molding and stacked with a cross-ply layup.

## 2. Methodology

The developed framework, EMMMA, presented in this study covers the micromechanical approach of an MSM process. The micromechanical modeling of MSM methods is often based on the concept of RVEs [19], similar to the framework in the current study. Utilizing an RVE model, the macromechanical properties of a material can be estimated [20]. Kanit et al. [21] emphasize the importance of a sufficiently large RVE that is statistically representative of the composite such that it includes enough information on the material’s micromechanical structure. However, the RVE must still be small enough to be considered a volume element of the continuum and comply with the separation of the different scales.

**Figure 1:**
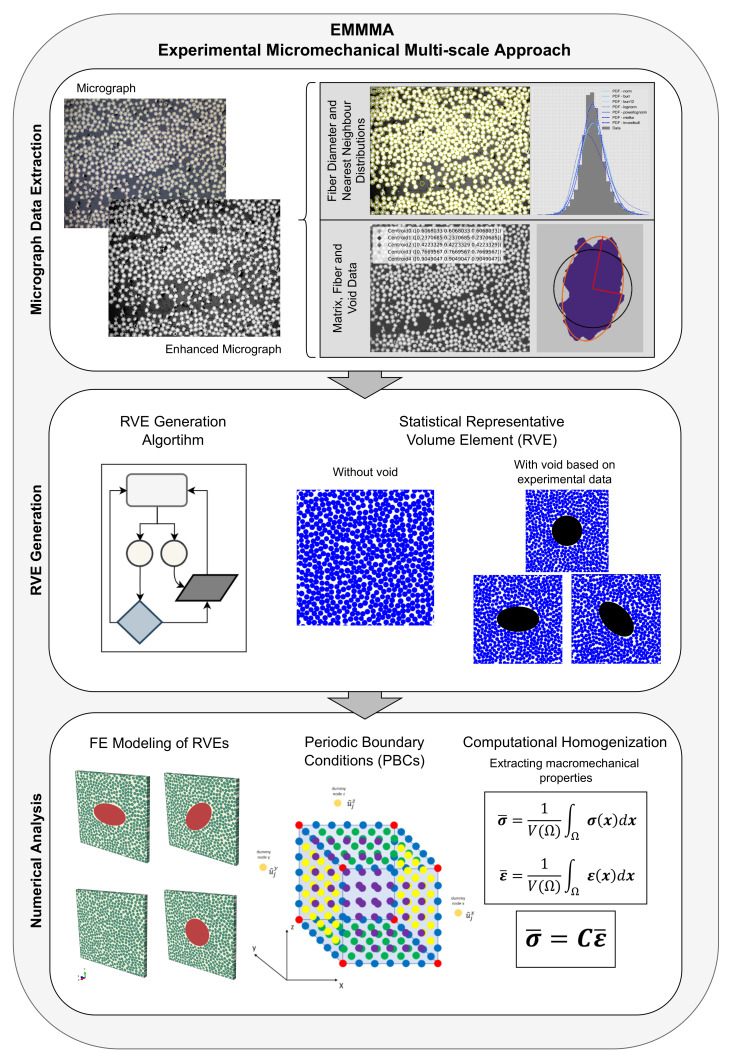
The workflow for the micromechanical simulation approach in a multi-scale modeling framework, referred to as EMMMA.

The workflow of the framework is presented in Figure 1. The framework consists of three main steps: the first step is the *Micrograph Data Extraction*, where micrographs from the material of interest are analyzed with image processing techniques to extract data on the matrix, fiber, and voids, used for the RVE generation. The second step is the *RVE Generation*, where data from micrographs are used in an algorithm to produce a periodic statistical RVE comparable to a real micromechanical structure of a UD composite material. The third and final step is the *Numerical Analysis*, where the Finite Element (FE) model is created and the macromechanical properties are computed using first-order computational homogenization.

The following sections will describe the different steps of the framework more thoroughly, and then, EMMMA will be applied to a real case.

### 2.1. Micrograph Data Extraction

The framework uses a periodic statistical RVE that is generated based on fiber distribution data extracted from actual micrographs of a UD CFRP material. This technique was introduced by Vaughan et al. [22], and EMMMA’s RVE generation algorithm applies the same methodology. The data extracted from the micrographs and used in the RVE algorithm are the first and second Nearest Neighbor (NN) distance distributions and the fiber diameter (${⌀}_{f}$) distribution. Volume fractions for the fibers ($V_{f}$), matrix ($V_{m}$), and voids ($V_{v}$) are also data that are extracted from the micrographs. The data are extracted from micrographs looking only in the fiber direction. Alternative approaches for microscopy are out of the scope for this study. Microscopy was selected due to its simplicity and accessibility.

#### 2.1.1. Image Processing

**Figure 2:**
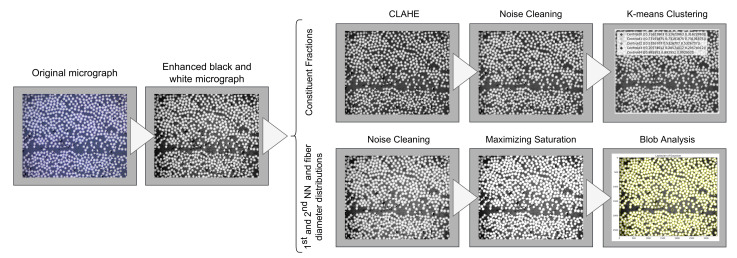
The imaging process workflow.

The micrographs are the foundation of the data used in the RVE generation algorithm. The constituent fractions are determined using quantitative optical microscopy, determining the area fractions in the micrographs. The area fractions are calculated from random selections of material sections and, therefore, also represent the constituents’ volume fractions [23]. The fiber distribution data are extracted utilizing different image processing techniques, and the micrographs are filtered to optimize the retrieved data depending on the sought data. The micrographs are first enhanced and converted to a black and white image, and then, in the case of retrieving constituent contents fractions, $V_{f}$ and $V_{m}$, the filtering consists of firstly enhancing the image contrasts in the micrograph using a Contrast-Limited Adaptive Histogram Equalization (CLAHE). Then follows a cleaning of noise surrounding the transitional edges between fiber and matrix using morphological dilation with an appropriate kernel size [24]. In the case of retrieving the fiber data, NN and ${⌀}_{f}$, the filtering consists of first removing fiber matrix noise, in the same process as that performed when retrieving constituent content fractions, and then, maximizing the image saturation. The image process is illustrated in Figure 2.

#### 2.1.2. Matrix, Fiber, and Void Data

Extracting the representative volume fractions of the constituents fiber and matrix is dependent on the quality of the micrographs. There are two techniques that can be used for this. The first is a K-means clustering technique [25,26], where the extraction is conducted on the enhanced and filtered micrographs. K-means clustering is a fast and simple algorithm to apply and, assuming appropriate filtering, returns representative fractional values of each constituent through clusters. The optimal number of clusters for a micrograph varies depending on the quality of the image; however, it is recommended to have between 3 and 7 clusters.

The K-means clustering technique is highly dependent on the quality of the micrographs, and an alternative method to extract fractions and, in particular, $V_{v}$, is to analyze the micrographs with a raster graphics editor [27]. The raster graphics editor is more time-consuming, but can also be used to extract void data, i.e., shape, size, and distribution of voids. With the help of the raster graphics editor, pixels representing a void can be selected and colored, and then further analyzed.

#### 2.1.3. Fiber Diameter and Nearest Neighbor Distributions

Extracting the fiber distribution data for the first and second NN, and ${⌀}_{f}$, the fiber tows in the micrographs are identified by utilizing a so-called Blob Detection Algorithm (BDA). The BDA utilizes a Laplacian of Gaussian (LoG) method [25,28,29], and the ${⌀}_{f}$ of each identified fiber tow, or blob, is calculated as a function of the standard deviation of the LoG function, σ, according to $r=\sqrt{2\sigma }$. The first and second NN distances are retrieved from the same identified fiber tows through unsupervised NN learning, using a ball tree structure [30].

**Figure 3:**
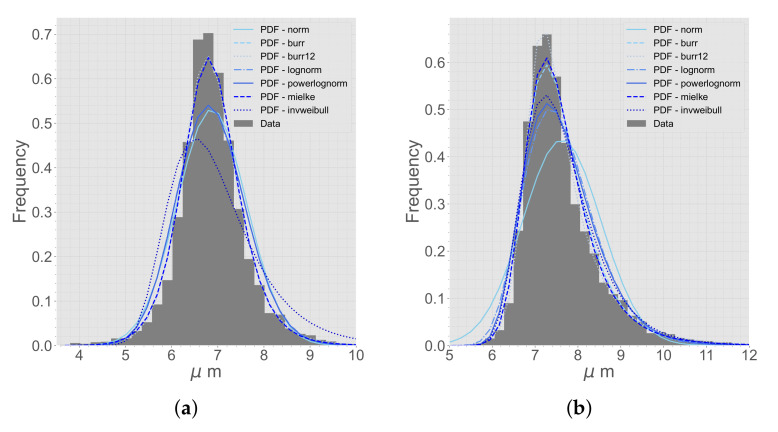
A selection of distribution functions fit to the extracted micrograph data. (**a**) First NN. (**b**) Second NN.

The data from the micrographs are compared to different distribution functions [31]. A selection of different distribution functions that are tested for the first and second NN can be seen in Figure 3. The best distribution function is decided based on a best fit from the Sum of Squares Error (SSE).

### 2.2. RVE Generation

Given the anisotropic nature of CFRP, the simplest form of RVE; with a regular periodic fiber arrangement, is often insufficient [32]. This type of fiber arrangement could underestimate, e.g., damage initiation or matrix failure [33]. Theoretical RVE algorithms generating a random fiber arrangement most often attempt to imitate the actual fiber distribution [34,35]. RVE modeling can also be based on copying the micromechanical characteristics found with, e.g., X-ray CT [36], which is more advanced and requires more expensive equipment compared to microscopy. Therefore, an experimentally based RVE generation algorithm, generating accurate fiber distributions based on data from the true micromechanical geometry extracted using 2D microscopy (Section 2.1), was used [22].

**Figure 4:**
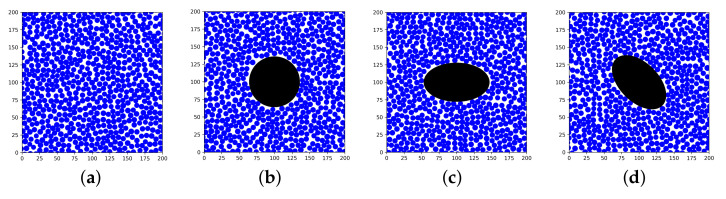
Periodic RVEs generated with the algorithm (**a**) without a void, (**b**) with a circular void, (**c**) with an elliptical void angled $0^{\circ }$, and (**d**) with an elliptical void angled $45^{\circ }$.

The RVE generation algorithm was implemented in Python, and the algorithm distributes the fibers periodically within a 2D area of LxL (Figure 4), where *L* is the RVE side length. For the RVE to represent a continuous body of a UD composite material, it needs to be repeatable in all directions, i.e., periodic. The RVE was only extended by 10 μm in the fiber direction since the undulations of fibers [37] were not considered in this study.

The addition of a void to the RVE was implemented in the RVE algorithm (Figure 4). The importance of the parameters void shape and void size and how they affect the mechanical properties have been highlighted in the literature [16,17]. True void shape could be implemented to find local initiation points [38]. However, since no void has the same shape, this could be time-consuming if needed to model all voids. Therefore, in this study, the voids were idealized with a circular or an elliptical shape and implemented based on a varying local void fraction, $V_{v}$, which is based on the extracted void data. The elliptical voids were angled $0^{\circ }$ and $45^{\circ }$. The voids were located in the center of the RVE and extruded in the fiber direction.

#### 2.2.1. Characterization of RVEs

Introducing defects, e.g., voids, in an RVE comes with trade-offs for the distribution of the fibers. A methodology by Pyrz [39,40] was utilized to analyze the RVE and characterize the pattern of fibers and the local interaction between fibers. All fiber centers in the composite material were considered as a spatial point pattern. Two statistical descriptors were utilized, and the first, a second-order intensity function, K(r), originally proposed by Ripley [41], is given by Equation (1).

(1) $$K\left(r\right)=\frac{A}{N^{2}}\sum_{k=1}^{N}w_{k}^{-1}l_{k}\left(r\right)$$

where *A* is the observation area, i.e., the area of the RVE, *N* the number of fibers in the observation area, $l_{k}\left(r\right)$ is the number of points within the radial distance *r* of the given fiber, and $w_{k}$ is a correction factor accounting for edge effects when the radial distance reaches outside of the observation area. $w_{k}$ is defined as the ratio of the circumference within the observation area to the whole circumference (2πr). The function is defined as the number of points expected to lie within the radial distance, *r*, of an arbitrary point (a fiber center), divided by the number of points per unit area, $N_{a}$.

The second statistical descriptor is the radial distribution function, describing the interaction between fibers locally. The radial distribution function is given by Equation (2).

(2) $$G\left(r\right)=\frac{A}{2\pi rN}\frac{dK(r)}{dr}$$

where dK(r) is the average number of points within a circle with inner radius *r* and outer radius dr.

While the radial distribution function, G(r), characterizes the occurrence intensity of inter-inclusion distances, the second-order intensity function, K(r), reflects quantitatively over the spatial arrangement as a function of distance from a fiber. Important for the modeling is the consideration of the voids in the correction factor, $w_{k}$. Two approaches are modeled analyzing the voids. In the first approach, the void is assumed to be part of the observation area, so it is disregarded in $w_{k}$. This is conducted for both elliptical and circular voids. In the second approach, conducted for only circular voids, the void is assumed not to be part of the observation area, and the circumference within the observation area is calculated with regard to the void. The circumference within the observation area is calculated according to the following criteria:

- if: $r_{void}>r$—remove the circumference within the void;
- if: $r_{void}<r$—remove the circumference of the void within the observation area.

The second-order intensity function, K(r), is compared to a pattern of Complete Spatial Randomness (CSR) (Equation (3)).

(3) $$K_{r}\left(r\right)=\pi r^{2}$$

This statistical analysis is not necessarily a part of EMMMA. The analysis was performed to see how voids might affect the fiber distribution of the RVE generation algorithm. This analysis was conducted in the case study for the generated RVEs.

### 2.3. Numerical Analysis

The numerical analysis was conducted in a commercial software used for finite-element analysis to solve the stresses and strains for the RVE. There is not yet a best practice on how to run an MSM approach, and it can be time-consuming to learn the process of new software, while the software might not be adapted to fit your specific needs [42]. There are available MSM techniques implemented in commercial software (e.g., Altair${}^{®}$ Multiscale Designer and Digimat from e-Xtreme Engineering); however, they are still maturing. Tchalla et al. [43] developed an ABAQUS toolbox that uses ABAQUS to execute an MSM analysis, showing that putting together your own framework streamlines the simulations and gives control of the different parts of the process. A part of the process is the RVE generation, and creating an RVE can be performed in many different ways [44], both using commercial software or developed plug-ins [45,46]. Spoonire et al. [47] highlight the importance of using large volumes and a sufficient number of fibers for the RVEs to obtain accurate results. Commercial software limits flexibility, and developing your own approach increases the options and control. Without a best practice, this study implemented its own FE approach and RVE generation because of the necessity to consider the experimental input based on the true micromechanical geometry.

#### 2.3.1. FE Modeling

The finite size of an RVE is important. As a starting point for the RVE size, *L*, a criterion from the study performed by Trias et al. [48], can be used as a guideline. Trias et al. defined a dimensionless variable δ relating to the RVE length, *L*, and to the fiber radius, *R*, as: $\delta =\frac{L}{R}$. For a typical CFRP material, the value of δ is said to be equal to 50. Using the mean value of the fiber radius extracted from the micrographs, a value of *L* can be obtained. In the case study, a sensitivity analysis for the size of the RVE is presented.

#### 2.3.2. Periodic Boundary Conditions

A Periodic Boundary Condition (PBC) was applied to the RVE model to fulfill the periodicity requirement. The displacements at the boundaries between adjacent elements need to be connected properly to avoid interpenetration or discontinuities. Confirming that the mesh for the FE RVE model is periodic, the equation for the displacements between two nodes on opposite surfaces of an RVE (i+,i−) is given by Equation (4) [49].

(4) $$u_{j}^{i+}-u_{j}^{i-}=\epsilon_{ij}^{0}l_{i}\;\left(i,j=x,y,z\right)$$

where $\epsilon_{ij}^{0}$ is the macro strain tensor and $l_{i}$ is the length of the RVE. If the length of an RVE is constant, $\epsilon_{ij}^{0}l_{i}$ can be exchanged with the displacement ${\hat{u}}_{j}^{i}$. A relative formulation was used, a decision based on the study by Garoz et al. [49], which emphasized the saved CPU time using the relative formulation instead of the absolute formulation. The PBCs were implemented using *EQUATION in ABAQUS and were connected to dummy nodes controlling the displacement difference between the two nodes, ${\hat{u}}_{j}^{i}$, where i,j=x,y,z. The PBCs were applied to the RVE model using a Python script run through ABAQUS.

#### 2.3.3. Computational Homogenization

Homogenization methods were employed to pass information in the multi-scale chain. It was assumed that the average mechanical properties of an RVE are equal to the average properties of the particular composite lamina. The framework developed utilizes a first-order computational homogenization scheme to find the effective macromechanical properties of the composite material [50]. The constitutive equation to be solved in matrix notation [51] can be seen in Equation (5).

(5) $$\bar{\sigma }=C\bar{\epsilon }$$

where C is the stiffness matrix and $\bar{\sigma }$ and $\bar{\epsilon }$ are the average global stress and strain values. The stress field of the RVE was determined through Finite-Element Analysis (FEA). From the FE simulation results, the average global values were computed as the volume average of the local stresses and strains of the RVE (Equation (6)).

(6) $$\begin{array}{c} {\bar{\epsilon }}_{ij}=\frac{1}{V}\int_{V}\epsilon_{ij}dV\;{\bar{\sigma }}_{ij}=\frac{1}{V}\int_{V}\sigma_{ij}dV \end{array}$$

where $\sigma_{ij}$ and $\epsilon_{ij}$ are the local strain and stress values of the RVE and *V* is the volume of the RVE.

Assuming the laminate is orthotropic, there are 9 unknown elastic constants to solve. These elastic constants are Young’s moduli $E_{x},E_{y},E_{z}$, Poisson’s ratios $\nu_{xy},\nu_{yz},\nu_{xz}$, and the three shear moduli $G_{xy},G_{yz},G_{xz}$. These can be solved through the compliance matrix, S, which is the inverse of the stiffness matrix ($S=C^{-1}$). Solving all nine unknown elastic constants for the material means applying six different load cases. The PBCs applied to the RVE model were altered applying a set of global strains such that $\epsilon_{ij}^{0}=\epsilon_{ij}$. The RVE length is constant, and therefore, the strain can be implemented as a displacement on the dummy nodes connected to the PBC. This constitutes a Boundary Value Problem (BVP) to be solved [3]. The computation was performed with a Python script run through ABAQUS. The full description of the matrix calculations is found in Appendix A.

## 3. Case Study

The framework, EMMMA, was implemented and verified on a real case based on a commercial composite material. In the case study, each step of the framework is presented.

### 3.1. Material System

**Figure 5:**
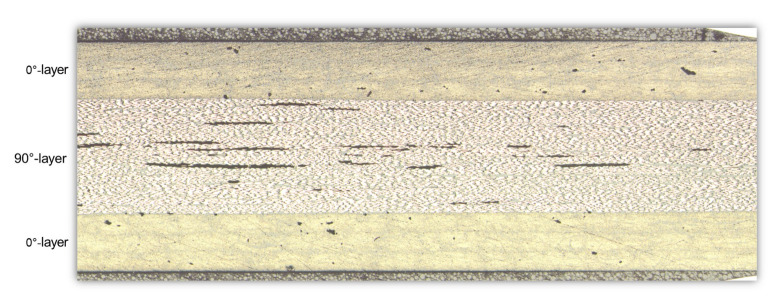
Full cross-section cut micrograph of the manufactured plate.

The material used in the case study was a UD CFRP prepreg. The UD tape has an epoxy matrix system 2300 from Toray [52]. It is a so-called “snap cure” matrix system with a cure time of 120 s in 130 ${}^{\circ }$C. According to the datasheet, the prepreg contains Toray T700S carbon fibers and has a $V_{f}$ of 0.58. The thickness of the UD tape in its uncured state is 0.22 mm. The investigated material system was prepared using heated compression molding with a pressure of 0.7 MPa. The material was manufactured as a plate with a size of 345 × 345 mm and a cross-ply layup sequence ${\left[0_{3}^{\circ }/90_{3}^{\circ }\right]}_{S}$. In Figure 5, a micrograph of the material is presented, showing a complete cross-section of the material. It can be observed that the voids were mainly elongated along the fiber direction ($90^{\circ }$-layer). The length of the voids varied between 100 μm and up to 1000 μm. This supports the idealization of the void as a cylinder when added to the RVE models.

### 3.2. Micrograph Data Extraction

Four microscopy specimens were prepared from varying positions of the manufactured plate. The micrograph samples were cut with a diamond blade to minimize surface damage and then polished with up to P4000 grit silicon carbide abrasive papers to reach an undamaged surface. To make a representative selection, two of the specimens were taken from the center of the plate and two were taken from an edge position of the plate. These positions represent two extreme positions regarding air evacuation during the manufacturing process. The micrographs were taken looking in the fiber direction. For each of the four micrograph samples, a series of four images were used, meaning 16 micrographs were used to generate the statistical data for the RVE generation algorithm. The micrographs covered areas with different and varying micromechanical geometry. The micrographs were acquired with an Olympus BX53 optical microscope with up to 50× magnification. The micrographs were taken with 5×, 20×, and 50× magnification.

#### 3.2.1. Matrix, Fiber, and Void Data

The volume fractions of the manufactured plate are presented in Table 1. A micrograph sample had an area of approximately 20 mm${}^{2}$ in the fiber direction. A micrograph taken with 50× and 20× magnification covers approximately 0.05 mm${}^{2}$, respectively 0.22 mm${}^{2}$. This means we would need 400, respectively 100 micrographs, to cover the complete surface of a micrograph sample. To determine the material’s void fraction, a cross-section of at least 80 % of the sample should be analyzed to have an error below 15 % [53]. Therefore, the void fraction, $V_{v}$, was decided only using the 5× magnification micrographs covering the complete surface of each sample. The fiber data for the RVE algorithm and the void data were extracted with 50× or 20× magnification micrographs.

The value of the 50× magnification micrographs is the average value of over 100 micrographs. The volume fractions for the 20× micrographs were calculated using the K-means algorithm, where the optimal amount of clusters were five. The quality of the micrographs were limited because of limited lighting in the microscope; therefore, voids in micrographs were clustered as matrix in the K-means algorithm, and the sum of $V_{m}$ and $V_{f}$ equaled 1. $V_{v}$ was calculated using a raster graphics editor, Photoshop^®^, together with the micrographs with 5× magnification.

**Figure 6:**
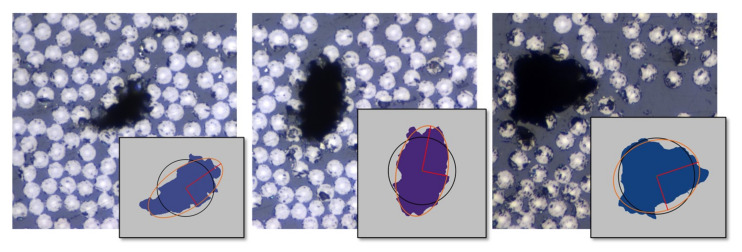
Comparing true voids from micrographs to a circular and an elliptical shape.

To have a better understanding of the effects of voids, it is of interest to evaluate their actual characteristics. The voids in the RVEs were based on actual extracted data from the micrographs of the CFRP material. The void data were extracted analyzing the micrographs with 50× magnification in a raster graphics editor. The shape of voids is almost never perfect. The shape varies in geometry and has local notches that could affect a crack formation scenario. Simplifying the void for an RVE model and extracting effective macromechanical properties, the overall shape that is suitable seems to be a circle or an ellipse. Figure 6 presents three different void shapes from a micrograph. The voids were slightly curved, elongated, or round. The circle and ellipse centers are located in the Center of Gravity (CoG) of the void. The elliptical shape is based on an assumption of equal normalized second central moments of the void and the ellipse. The circle is adapted based on the assumption that the circle has the same area as the void. After the shape fit, the ellipse covered the void better in most cases and was adapted when the void was shaped more like a circle.

**Figure 7:**
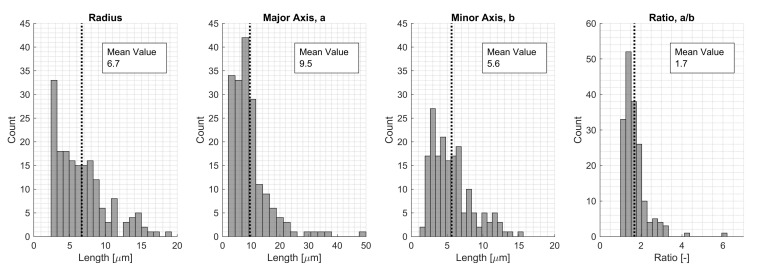
Extracted void data for the manufactured plate looking at shape parameters for a fitted circular and elliptical void shape.

The shape comparison was conducted for a great number of voids based on extracted void data from the 50× magnification micrographs. In Figure 7, histograms of the predicted circle and ellipse geometries are presented. The fitted circle had a mean void radius of 6.7 μm, and the largest voids were represented by a radius of up to 20 μm. The same data for the ellipse fit are presented looking at the major and minor axes lengths. The average ratio of the major and minor axes was 1.7, meaning most of the voids were elliptical and not circular. The average length of the major and minor axes was 9.5 μm resp. 5.6 μm. However, for an ellipse, the largest major axis was 50 μm. This would represent a void with a total length of 100 μm, which was more than twice as large as the largest radius for a fitted circular void. The average values were used in this study to represent typical scenarios.

**Figure 8:**
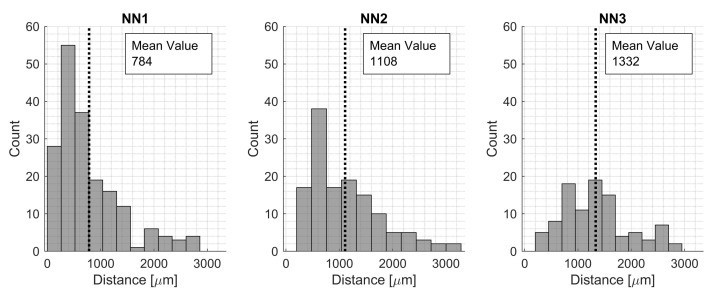
Presenting the first, second, and third NN distances between voids for the manufactured plate.

Using an RVE size of 200 μm, it was also of interest to see how close the voids were located to each other and if it was reasonable to model only one void within an RVE. First, second, and third neighboring voids were located, and the average distances were 784 μm, 1106 μm resp. 1332 μm (Figure 8). It was seen that some voids were located more closely than the chosen RVE size of 200 μm; however, using the average values to represent typical scenarios, modeling more than one void was not within the scope of this study. Only one void was investigated, which fit well with the mean values of the NN for the voids.

#### 3.2.2. Fiber Diameter and Nearest Neighbor Distributions

**Figure 9:**
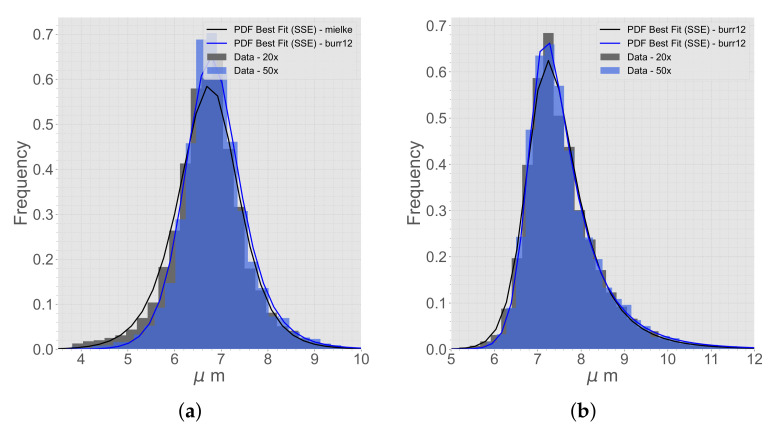
The best distribution functions of (**a**) first NN and (**b**) second NN, comparing the data from micrographs with 20× and 50× magnification.

**Figure 10:**
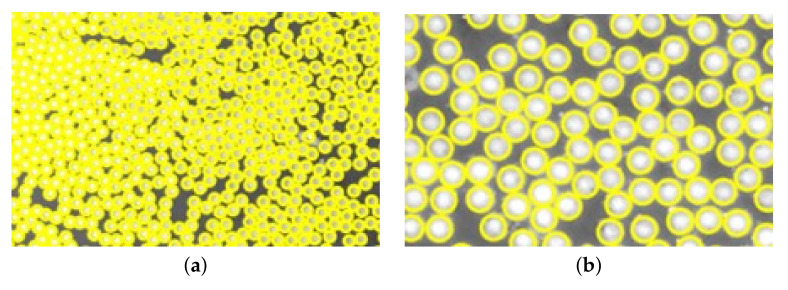
Blob analysis for (**a**) 20× magnification, and (**b**) 50× magnification micrographs.

The micrographs were analyzed with the BDA, and the best distribution functions for first and second NN were decided. The distribution functions extracted from the micrographs can be seen in Figure 9. The main difference between the magnifications, 50× and 20×, was the amount of data. The average amount of fibers in a micrograph with 50× magnification was approximately 800, compared to 3400 fibers for the 20× magnification micrographs. Even though there were more data for the 20× magnification micrographs, the data from the 50× magnification micrographs were preferred and used. Looking at the difference in the 20× and 50× magnification micrographs, the blob analysis was clearer for the 50× magnification micrographs (Figure 10). The blob analysis was not optimal for generating a distribution for ${⌀}_{f}$, and instead, a manual measurement of 50 fibers was conducted. The manual measurement showed that ${⌀}_{f}$ varied between 6.1 μm and 7.6 μm and the average size was 6.9 μm. These manually measured data were used in the RVE algorithm. The distribution functions and process parameters were calibrated such that these results corresponded correctly.

### 3.3. RVE Generation

The finite RVE size, *L*, is an important parameter. Following the rule of thumb given by Trias et al. [48], choosing the RVE size, *L*, according to $\delta =\frac{L}{R}$, where δ equals 50, and *R*, the average fiber radius, 3.45 μm, the formula gave a value of 172.5 μm as *L*.

**Figure 11:**
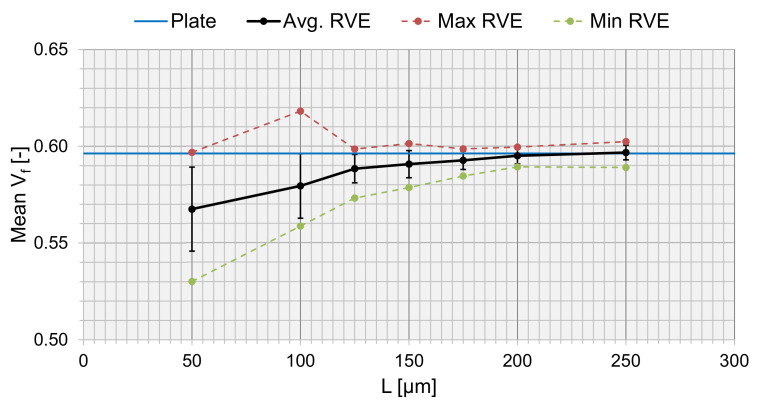
The fiber volume fraction, $V_{f}$, as a function of RVE side length, *L*.

To verify the choice of *L*, a sensitivity analysis was conducted. Ten RVEs each were generated for different sizes of *L*, and the average $V_{f}$ was extracted (Figure 11). $V_{f}$ converged with an increasing *L*, and was matched towards the true value of $V_{f}$ from Table 1 ($V_{f}=0.5963$, for the 50× micrographs). The value of $V_{f}$ from the generated RVEs corresponded well at L = 200 μm towards the true fiber volume fraction of the plate. The max and min values converged at higher RVE sizes, and the standard deviation decreased, meaning that the local varieties in the RVE did not matter as much for larger RVEs because the size of the RVE compensated for the overall fraction. However, the size of the RVE shall represents the micro-scale and should then be limited by the thickness of a lamina (220 μm). This fits well with the converged value of *L* = 200 μm.

**Figure 12:**
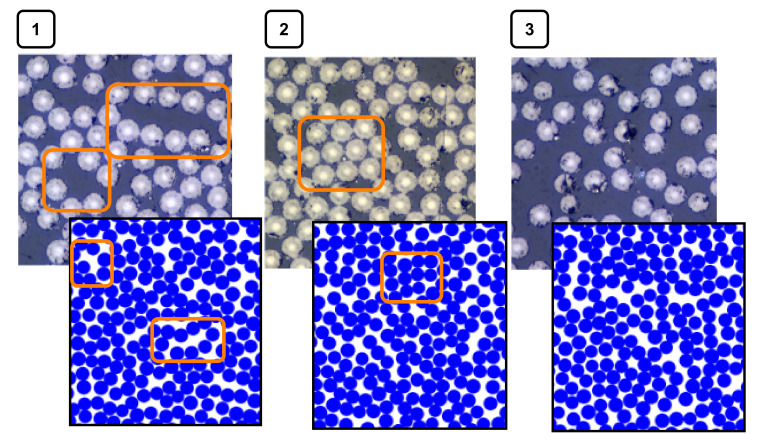
Comparison of actual microstructure and the generated RVE fiber distribution. The different images show (**1**) how smaller-matrix-dominating areas are captured, (**2**) how areas with tightly packed fibers are captured, and (**3**) how sparsely scattered fibers are difficult to capture.

The RVE size will depend on what you want to investigate, and local effects could be significant. The RVE algorithm was rather good at imitating and capturing some real events from the micrographs. In Figure 12, some events in an actual micrograph are compared with the generated RVE fiber distribution. It was also seen that sparsely scattered fibers, which could occur between plies, were not captured. Moving forward in the case study an RVE length, *L*, of 200 μm was used to represent the material.

Analyzing the voids of the manufactured plate, both circular and elliptical voids were generated in the RVEs varying the local void fraction. The area was kept constant between the circular and the elliptical void to be able to compare the results. For each void type, nine different void areas, $V_{A}$, and RVEs were generated. The ratio of the elliptical void axes was set to the mean value from Figure 7, which was 1.7. The sizes of the voids were determined and rounded to match the given area as well as possible. $V_{v}$ and $V_{f}$ for each generated RVE are summarized in Table 2, Table 3 and Table 4. $V_{f}$ is presented in two ways, where the first calculation considered the void, $V_{v}+V_{f}+V_{m}=100\%$, and the second did not consider the void, $V_{f}+V_{m}=100\%$. Two different calculations of $V_{f}$ were used to analyze how the void affected the surrounding fiber distribution.

#### Statistical Characterization of RVEs

**Figure 13:**
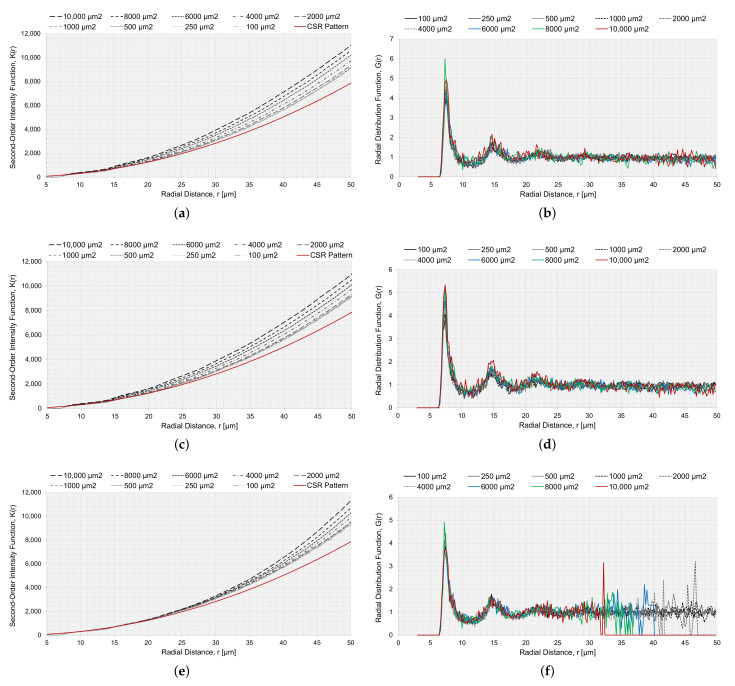
Second-order intensity function K(r) and the radial distribution function G(r) for RVEs with different $V_{A}$. (**a**) K(r) with the void as a part of the observation area for circular voids, (**b**) G(r) with the void as a part of the observation area for circular voids (**c**) K(r) with the void as a part of the observation area for elliptical voids angled $0^{\circ }$, (**d**) G(r) with the void as a part of the observation area for elliptical voids angled $0^{\circ }$, (**e**) K(r) with the void not as a part of the observation area and adjusting the correction factor $w_{k}$ accordingly for circular voids, and (**f**) G(r) with the void not as a part of the observation area for circular voids.

**Figure 14:**
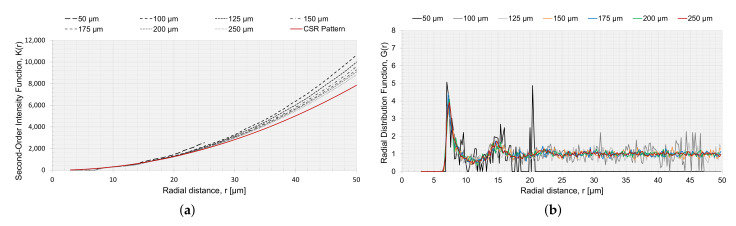
Second-order intensity function K(r) and the radial distribution function G(r) for different RVEs with side length *L* and no voids, (**a**) K(r) and (**b**) G(r).

The RVEs generated with a circular and elliptical void angled in $0^{\circ }$ were analyzed with the statistical descriptors K(r) and G(r), to characterize the spatial point patterns of the fiber distributions. K(r) for RVEs with voids is presented in Figure 13a,c,e. In Figure 13a,c, the voids were not considered in the correction factor $w_{k}$ (Section 2.2.1), and in Figure 13e, the circular void was considered in the correction factor $w_{k}$. If the void was not considered in the correction factor $w_{k}$, the small voids had slightly reduced values and the result tended to move together with an increased value for *r*. Comparing the point fields to a CSR pattern, all curves moved away from the CSR pattern function, indicating long-range clustering. A reduced $V_{f}$ should have a higher K(r) [35], and this was supported by the results for K(r) since a larger void decreased $V_{f}$, and the K(r) curves for larger void diverged slightly more than for the smaller voids. The same trend, but intensified, was observed for the approach where the void was part of the observation area. The same goes for the RVE size, *L* (Figure 14a). The smaller the RVE, the more $V_{f}$ is reduced (Figure 11). The curves for K(r) are very smooth, for both void size and RVE size, which indicated that the generated algorithm avoids regular positioning.

The radial distribution function, Figure 13b,d,f and Figure 14b, had a clear peak for all cases at approximately r=7, which correlated to the first NN distance (Figure 9a). The fluctuations of the function converged to a unity of 1. The larger fluctuations were connected to the lower $V_{f}$. Relating the first peak of the radial distribution and the height of the first peak to $V_{f}$, a higher peak had a reduced $V_{f}$. For the radial distribution function, the limitation was the size of the RVE, and the function would not converge to unity if the radial distance from a fiber was outside the observation area; therefore, when the radial distance was too large, it was no longer within the observation area, and the function G(r) became zero (Figure 14b).

### 3.4. FE Modeling

**Figure 15:**
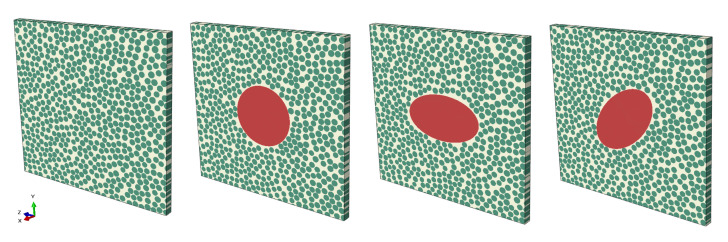
Illustrating the FE RVE models with no void or circular or elliptical voids.

The stress and strain analysis of the RVE models was conducted using the FE software ABAQUS 2019 [54], and the FE modeling was conducted in the pre-processor software ANSA [55]. The RVEs were modeled as plates (Figure 15) with linear elastic and isotropic material for the matrix and transverse isotropic properties for the fibers [56]. Typical material constants for carbon fibers were considered together with the properties reported by the datasheet of the material [52]. For the transverse isotropic properties of the carbon fibers, the longitudinal elastic modulus was 230 GPa, the transverse elastic modulus was 12.0 GPa, the in-plane Poisson ratio was 0.17, the out-of-plane Poisson ratio was 0.3, the in-plane shear modulus was 9.85 GPa, and the out-of-plane shear modulus was 4.62 GPa. The isotropic matrix’s elastic modulus and Poisson’s ratio were, respectively, 4.40 GPa, and 0.3. The interface between fiber and matrix was implemented as a perfect bond. The RVE was modeled in 3D with C3D8I (cube) elements and adjusted with C3D6 (wedge) elements to stay true to the geometry. The distance between two fibers was set to a minimum of 0.2 μm, which is approximately 6% of the fiber radius. References refer to this distance being somewhere in between 2 and 7 % of the fiber radius [35,57]. The minimum distance was needed such that it was still possible to model the space in the FE model with a couple of elements. The mesh size was set to 0.75 μm, giving a reasonable computational time, and it was ensured that the computational homogenization results for the given mesh size converged. The voids were modeled with 3D elements and implemented with a stiffness value of $10^{-6}$ MPa and a density value of $10^{-20}$$\frac{\mathrm{kg}}{m^{3}}$.

### 3.5. Results of the Case Study

#### 3.5.1. Prediction of Macromechanical Properties

**Figure 16:**
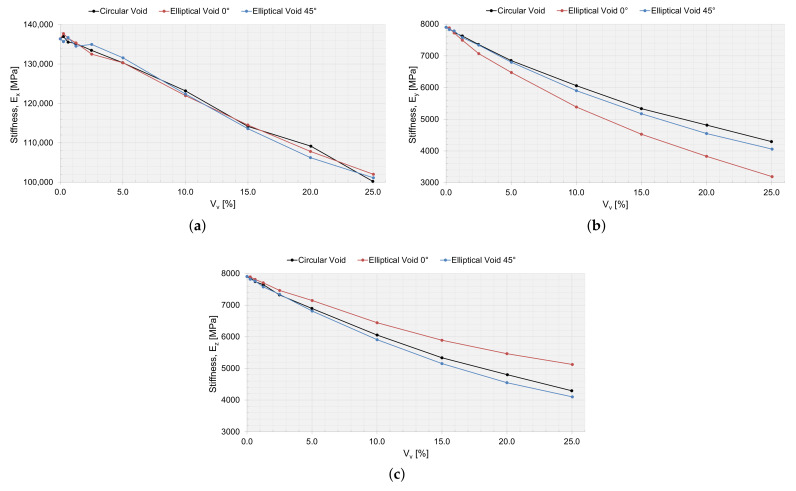
The effective macromechanical properties: (**a**) $E_{x}$, (**b**) $E_{y}$, and (**c**) $E_{z}$, as a function of void area, $V_{v}$, for a 200 μm RVE.

**Figure 17:**
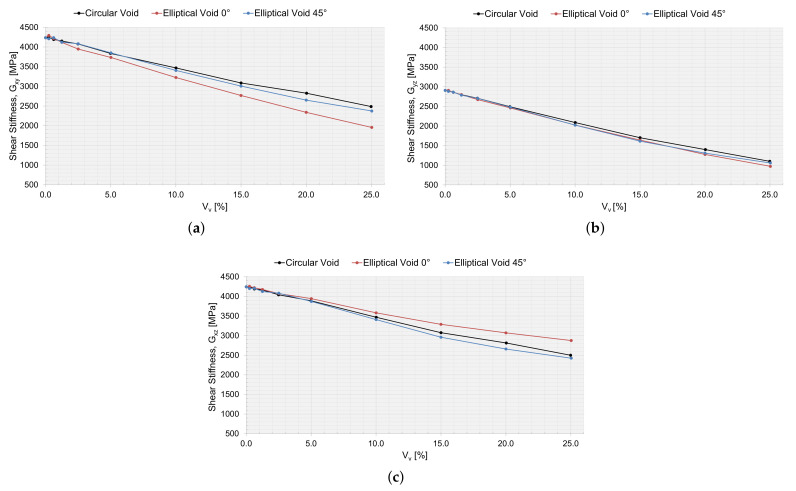
The effective macromechanical properties: (**a**) $G_{xy}$, (**b**) $G_{yz}$, and (**c**) $G_{zx}$, as a function of void area, $V_{v}$, for a 200 μm RVE.

**Figure 18:**
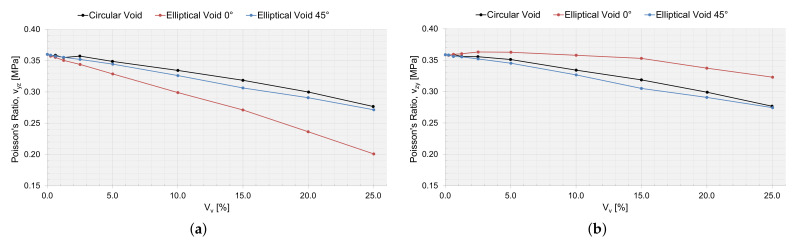
The effective macromechanical properties: (**a**) $\nu_{yz}$ and (**b**) $\nu_{zy}$, as a function of void area, $V_{v}$, for a 200 μm RVE.

Utilizing the first-order computational homogenization scheme, the effective macromechanical properties were calculated. In Table 5, the macromechanical properties are presented for the RVE simulation, without a void, and with an RVE size of 200 μm. The macromechanical properties for an RVE with a void and as a function of $V_{v}$ are presented in Figure 16, Figure 17 and Figure 18.

The drop in stiffness and shear stiffness was evident with an increasing $V_{v}$ (Figure 16 and Figure 17), and this could also be related to a decreasing $V_{f}$ for the RVEs (Table 2, Table 3 and Table 4). $E_{x}$ seems not to have been affected by the shape of the void and behaved equally with an increasing $V_{v}$. $E_{y}$ and $E_{z}$ were the same for circular voids and elliptical voids angled $45^{\circ }$. The elliptical void angled $0^{\circ }$, had a higher stiffness in the same direction as the major axis. The shear stiffness was not affected if the void was circular or elliptical angled $45^{\circ }$. Only the Poisson ratios $\nu_{yz}$ and $\nu_{yz}$ were affected when adding a void. The voids decreased the ratio of change in width and change in length, more or less so for an elliptical void angled $0^{\circ }$, depending on if the direction of the major axis was orthogonal or coincided with the loading direction.

In the numerical model, the void shape was limited and idealized using two shapes, circular and elliptical. The void was centrally located, and only one void in an RVE was implemented. The model covers elongated voids, and if one was to analyze spherical voids, a full 3D RVE model needs to be modeled. This implementation was simplified to suit an industrial implementation, but with a modular approach, each part is possible to develop even further.

#### 3.5.2. Verification with Static Testing

A static tensile test was performed for two specimens of the manufactured cross-ply plate to determine the Ultimate Tensile Strength (UTS) and stiffness. The test was performed according to ASTM D3039/D3039M-17 [58] on a static testing machine, Instron 4505, with a calibrated 100 kN load cell. The displacement rate was set to 2 mm/min. The strain was measured using a Digital Image Correlation (DIC) system, GOM Aramis 5M LT, with a 50 mm lens. The Aramis system recorded the strain field on one surface of the specimens during testing, and the results were used for calculating the stiffness. The specimen geometry was inspired by [59] for manufacturing purposes, and glass fiber tabs (50 mm long) were used. The tabs had a rough surface for better gripping. The specimen geometry was adjusted and manufactured in accordance with ASTM D3039/D3039M-17 [58]. The tensile test results are presented in Table 6. The framework extracted the macromechanical properties for a UD laminae; therefore, the static test results for the cross-ply laminate were compared to $E_{x}$ and $G_{xy}$ calculated analytically with Classical Lamination Theory (CLT). The input for the CLT calculation was the extracted macromechanical properties from the computational homogenization procedure (Figure 16, Figure 17 and Figure 18). The properties $E_{x}$ and $G_{xy}$ as a function of void area, $V_{v}$, for a cross-ply were calculated and are presented in Table 7. The results were in the same order of magnitude as the testing results.

## 4. Discussion and Conclusions

A framework covering the micromechanical approach of an MSM process was developed, answering the need to add manufacturing defects, i.e., voids, and their true statistical variability. The framework showed good predictions of the macromechanical properties, compared and verified with static testing. The macromechanical properties, weak spots, and critical areas can be determined by using several RVEs with a varying local void fraction in a full-scale macro-model. The framework is also flexible with the possibility to exchange the analyzed material with another by adjusting the input data. Without adding too many details, the simulation cost was reasonable and the approach showed an excellent possibility of identifying critical areas of a composite structure if connected to a complete MSM approach. There is also the possibility to expand the analysis by looking further into the micromechanics and specific damage modes.

### 4.1. RVE Generation

Capturing the typical micromechanical geometry of a CFRP material is essential. This is supported by the experimental approach used in this study, showing that the size of the RVE mattered if the aim was to capture a true $V_{f}$, which, in turn, highly affects the macromechanical properties. If voids were included in the analysis, varying the local void fraction, $V_{v}$, the fiber fraction, $V_{f}$, was slightly reduced, but not to such an extent that the fiber distribution would no longer be valid. Even the large voids still generated an approved surrounding fiber distribution for the chosen RVE size (Table 2, Table 3 and Table 4).

The appropriate size of an RVE, generated with the algorithm in this study, was clearly seen analyzing $V_{f}$ (Figure 11). The guideline introduced by Trias et al. [48] corresponded well to the true $V_{f}$; however, a sensitivity analysis would be recommended to find the perfect fit. The chosen RVE represented well the material that was analyzed. The RVE generation algorithm captured the fiber distribution also corresponding to a true fiber distribution compared to real micrographs. The rule of thumb from Trias et al. might not be valid if voids are present in the RVE; however, to address this, the micromechanical model needs to be connected to a macro-scale model. Then, an evaluation of the effect the size of an RVE with voids might have on the macromechanical response can be conducted. It would be essential to consider the actual statistical void data regarding the shape, size, distribution, and fraction of the voids.

### 4.2. Implementation of Voids

After finding a representative size of the RVE, the local void fraction was varied, including the extracted experimental void data. If one RVE were to represent the material maintaining the true void volume fraction, $V_{v}=0.0062$, the RVE size would need to be incredibly large to represent the largest void size. No material has only one void size. A large RVE will increase the computational time, and the RVE would be too large to represent the micromechanical scale of the material.

Illustrating the issue, doubling the RVE size and maintaining a void volume fraction of $V_{v}=0.0062$, the RVE size would now represent the thickness of two laminae, moving away from the micro-scale representation. The RVE was modeled with a circular void (${⌀}_{v}$ = 35.6 μm) and an elliptical void angled $0^{\circ }$ (major axis, a = 23.2 μm, minor axis, b = 13.6 μm). These voids represent the larger voids found in the material, but the numerical results for the effective macromechanical properties showed a very small difference. Table 8 presents the percentual difference from a baseline simulation (200 μm RVE without a void) for two different sizes of RVE using a void volume fraction of 0.0062, and the maximum difference was below 3%. This difference could represent a numerical error or be connected to the variation in fiber volume fraction from the random RVE generation. To find local varieties in the material properties, the local void volume fraction needs to be considered. The RVE needs to be utilized, not only as an overall representation of the material, but also for a local variation of the material properties. The local void fraction varies throughout a material and depends on geometry and manufacturing techniques.

When varying the local void volume fraction and the size of the voids in an RVE, the size is an important factor. The void fraction of the manufactured plate studied was 0.0062, which corresponded to a void area of 250 μm${}^{2}$. For this particular void fraction, the void size for the circular void (radius of 8.9 μm) and the elliptical void (major axes of 11.6×6.8μm) was still larger than the average values of the extracted void data (Figure 7). However, the macromechanical properties were slightly affected by these small voids in the RVE (Figure 16, Figure 17 and Figure 18) and the laminate (Table 7). The largest void simulated ($V_{v}=0.25$) reduced the elastic properties in the RVE by approximately 25% for $E_{1}$, up to 59% for $E_{2}$ and up to 48% for $E_{3}$. The larger voids might not frequently occur in the true laminate; however, the results show that it is important to consider the void statistics of the true material when moving to the macromechanical modeling, not missing the effects of a local void fraction and the effect that large voids might have on the material properties.

Purslow [60] ranks composite material quality based on the void fraction. A composite material with $V_{v}>5,\%$ is ranked as having *very poor* quality. For $V_{A}$ = 2000 μm${}^{2}$, the numerical results for the laminate corresponded well to the static testing results (Table 7), and the corresponding local $V_{v}$ was 5.59%; thus, the quality of the composite would have been ranked as *very poor*. For an elliptical void shape, the corresponding void size for the void fraction of 5.59% was 32.9 μm × 19.4 μm, and this void size existed in a laminate with a $V_{v}$ of 0.62% (Figure 7). However, the laminate with a void fraction of 0.62%, would be graded as having *good* quality ($0.5\%<V_{v}\leq 1\%$). This phenomenon needs to be considered if implemented in an MSM approach, for example using a statistical description of the size, shape, distribution, and fraction of the voids. Keep in mind that the test results and numerical results include measurement errors that limit a direct comparison; however, the comparison can highlight important aspects.

The void shape was important if connected to the size of the voids. A representative circular void area will have a much smaller radius than the void’s actual size, which corresponds better to the elliptical shape. Depending on what damage you might want to consider in your model, the void shape will matter [38]. The study did not consider the correlation between the void’s diameter and length since the RVE would need to be even larger to keep a similar correlation for the fibers.

## Figures and Tables

**Table 1:** Average volume fractions for fiber ($V_{f}$), matrix ($V_{m}$), and void ($V_{v}$) of the manufactured plate.

Magnification	Avg. $V_{f}$ (-)	Avg. $V_{m}$ (-)	Avg. $V_{v}$ (-)
5	N/A	N/A	0.0062
20	0.5808	0.4192	N/A
50	0.5963	0.4037	N/A

**Table 2:** $V_{v}$ of the RVEs with a circular void and the resulting $V_{f}$.

$V_{v}$ (-)	${⌀}_{v}$ (μm)	Area (μm${}^{2}$)	$V_{f}$ (-)	$V_{f}$ (-) (w.o. Void)
0.2498	112.8	10,000	0.4341	0.5787
0.2003	101.0	8000	0.4757	0.5949
0.1500	87.4	6000	0.4943	0.5815
0.1001	71.4	4000	0.5300	0.5890
0.0499	50.4	2000	0.5656	0.5953
0.0249	35.6	1000	0.5751	0.5898
0.0125	25.2	500	0.5852	0.5925
0.0062	17.8	250	0.5880	0.5917
0.0025	11.2	100	0.5958	0.5972

**Table 3:** $V_{v}$ of the RVEs with an elliptical void angled $0^{\circ }$ and the resulting $V_{f}$.

$V_{v}$ [-]	Major Axis, a (μm)	Minor Axis, b (μm)	Area (μm${}^{2}$)	$V_{f}$ (-)	$V_{f}$ (-) (w.o. Void)
0.2503	73.6	43.3	10,000	0.4420	0.5896
0.2000	65.8	38.7	8000	0.4675	0.5844
0.1500	57.0	33.5	6000	0.4945	0.5818
0.1001	46.5	27.4	4000	0.5273	0.5859
0.0501	32.9	19.4	2000	0.5657	0.5956
0.0251	23.3	13.7	1000	0.5767	0.5915
0.0125	16.4	9.7	500	0.5870	0.5945
0.0062	11.6	6.8	250	0.5912	0.5949
0.0025	7.4	4.3	100	0.5938	0.5953

**Table 4:** $V_{v}$ of the RVEs with an elliptical void angled $45^{\circ }$ and the resulting $V_{f}$.

$V_{v}$ (-)	Major Axis, a (μm)	Minor Axis, b (μm)	Area (μm${}^{2}$)	$V_{f}$ (-)	$V_{f}$ (-) (w.o. Void)
0.2503	73.6	43.3	10,000	0.4383	0.5847
0.2000	65.8	38.7	8000	0.4583	0.5728
0.1500	57.0	33.5	6000	0.4899	0.5763
0.1001	46.5	27.4	4000	0.5305	0.5895
0.0501	32.9	19.4	2000	0.5692	0.5993
0.0251	23.3	13.7	1000	0.5850	0.6000
0.0125	16.4	9.7	500	0.5830	0.5903
0.0062	11.6	6.8	250	0.5959	0.5996
0.0025	7.4	4.3	100	0.5868	0.5882

**Table 5:** Average effective macromechanical properties for an RVE without a void.

Property	Avg. Value
$E_{x}$	136,395 MPa
$E_{y}$	7900 MPa
$E_{z}$	7904 MPa
$G_{xy}$	4235 MPa
$G_{yz}$	2908 MPa
$G_{xz}$	4244 MPa
$\nu_{xy}$	0.22
$\nu_{yx}$	0.01
$\nu_{xz}$	0.22
$\nu_{zx}$	0.01
$\nu_{yz}$	0.36
$\nu_{zy}$	0.36

**Table 6:** Static tensile test results of the manufactured cross-ply plate with $V_{f}=0.5963$ and $V_{v}=0.0062$.

UTS (MPa)	Stiffness (MPa)
1377	68,519
1268	67,368

**Table 7:** Analytical results calculated with CLT for a cross-ply laminate based on the RVE results.

$V_{v}$ (-)	Circular Void	Circular Void	Elliptical Void $0^{\circ }$	Elliptical Void $0^{\circ }$	Elliptical Void $45^{\circ }$	Elliptical Void $45^{\circ }$
	$E_{x}$ (MPa)	$G_{\mathrm{xy}}$ (MPa)	$E_{x}$ (MPa)	$G_{\mathrm{xy}}$ (MPa)	$E_{x}$ (MPa)	$G_{\mathrm{xy}}$ (MPa)
0.25	52,352	2485	52,677	1956	52,687	2374
0.20	57,087	2825	55,909	2339	55,490	2649
0.15	59,858	3085	59,624	2768	59,495	3007
0.10	64,753	3467	63,790	3224	64,265	3403
0.05	68,734	3835	68,528	3733	69,320	3850
0.025	70,567	4078	69,933	3948	71,309	4081
0.0125	71,573	4149	71,585	4118	71,200	4119
0.006	71,806	4191	72,287	4217	72,419	4236
0.0025	72,580	4239	72,960	4294	71,912	4207
0	72,308	4235	72,308	4235	72,308	4235

**Table 8:** Comparison of the average effective macromechanical properties maintaining the void volume fraction. The 200 μm RVE without a void is considered as the baseline. Only Young’s modulus and the shear modulus are presented; due to a very low variation, Poisson’s ratio is not included.

Property	RVE 200 μm No Void (Baseline)	RVE 200 μm Circ. Void	RVE 400 μm Circ. Void	RVE 200 μm Ellip. Void	RVE 400 μm Ellip. Void
$V_{v}$ (-)	0	0.0062	0.0062	0.0062	0.0062
Void size	-	${⌀}_{v}$ = 17.8 μm	${⌀}_{v}$ = 35.6 μm	*a* = 11.6 μm *b* = 6.8 μm	*a* = 23.2 μm *b* = 13.6 μm
$V_{f}$ (-)	0.5902	0.5880 (incl. void)	0.5965 (incl. void)	0.5912 (incl. void)	0.5920 (incl. void)
$E_{x}$ (MPa)	136,395	−0.61%	+0.83%	+0.10%	−0.48%
$E_{y}$ (MPa)	7900	−2.11%	−1.09%	−2.24%	−2.59%
$E_{z}$ (MPa)	7904	−2.02%	−1.23%	−1.13%	−1.35%
$G_{xy}$ (MPa)	4235	−1.04%	0.50%	−0.43%	−1.02%
$G_{yz}$ (MPa)	2908	−1.65%	−0.89%	−1.51%	−1.62%
$G_{xz}$ (MPa)	4244	−1.32%	+0.02%	−0.57%	−0.94%

## Data Availability

Not applicable.

## Abbreviations

The following abbreviations are used in this manuscript:

BDA	Blob Detection Algorithm
BEVs	Battery Electric Vehicles
BVP	Boundary Value Problem
CFRP	Carbon-Fiber-Reinforced Plastics
CLAHE	Contrast-Limited Adaptive Histogram Equalization
CLT	Classical Lamination Theory
CoG	Center of Gravity
CSR	Complete Spatial Randomness
DIC	Digital Image Correlation
FE	Finite Element
FEA	Finite-Element Analysis
LoG	Laplacian of Gaussian
MSM	Multi-Scale Modeling
NN	Nearest Neighbor
PBC	Periodic Boundary Condition
RVE	Representative Volume Element
SMC	Sheet Molding Compounds
SSE	Sum of Square Error
UD	Unidirectional
UTS	Ultimate Tensile Strength

## Appendix A. Computational Homogenization

This Appendix covers the expansion of the matrix notation for the first-order computational homogenization scheme presented in the study. The constitutive equation to be solved in full matrix notation [51] can be seen in Equation (A1).

(A1) $$\left[\begin{array}{c} {\bar{\sigma }}_{11}\\ {\bar{\sigma }}_{22}\\ {\bar{\sigma }}_{33}\\ {\bar{\tau }}_{12}\\ {\bar{\tau }}_{23}\\ {\bar{\tau }}_{13} \end{array}\right]=\left[\begin{array}{cccccc} C_{11} & C_{12} & C_{13} & C_{14} & C_{15} & C_{16}\\ C_{21} & C_{22} & C_{23} & C_{24} & C_{25} & C_{26}\\ C_{31} & C_{32} & C_{33} & C_{34} & C_{35} & C_{36}\\ C_{41} & C_{42} & C_{43} & C_{44} & C_{45} & C_{46}\\ C_{51} & C_{52} & C_{53} & C_{54} & C_{55} & C_{56}\\ C_{61} & C_{62} & C_{63} & C_{64} & C_{65} & C_{66} \end{array}\right]\left[\begin{array}{c} {\bar{\epsilon }}_{11}\\ {\bar{\epsilon }}_{22}\\ {\bar{\epsilon }}_{33}\\ {\bar{\gamma }}_{12}\\ {\bar{\gamma }}_{23}\\ {\bar{\gamma }}_{13} \end{array}\right]$$

where C is the stiffness matrix and ${\bar{\sigma }}_{ij}$ and ${\bar{\epsilon }}_{ij}$ are the average global stress and strain values. Assuming the laminate is orthotropic, there are nine unknown elastic constants to solve. They can be solved through the compliance matrix, S (Equation (A2)), which is the inverse of the stiffness matrix ($S=C^{-1}$).

(A2) $$S=\left[\begin{array}{cccccc} S_{11} & S_{12} & S_{13} & 0 & 0 & 0\\ S_{21} & S_{22} & S_{23} & 0 & 0 & 0\\ S_{31} & S_{32} & S_{33} & 0 & 0 & 0\\ 0 & 0 & 0 & s_{44} & 0 & 0\\ 0 & 0 & 0 & 0 & s_{55} & 0\\ 0 & 0 & 0 & 0 & 0 & s_{66} \end{array}\right]=\left[\begin{array}{cccccc} \frac{1}{E_{x}} & -\frac{v_{yx}}{E_{y}} & -\frac{v_{zx}}{E_{z}} & 0 & 0 & 0\\ -\frac{v_{xy}}{E_{x}} & \frac{1}{E_{y}} & -\frac{v_{zy}}{E_{z}} & 0 & 0 & 0\\ -\frac{v_{xz}}{E_{x}} & -\frac{v_{yz}}{E_{y}} & \frac{1}{E_{z}} & 0 & 0 & 0\\ 0 & 0 & 0 & \frac{1}{G_{xy}} & 0 & 0\\ 0 & 0 & 0 & 0 & \frac{1}{G_{yz}} & 0\\ 0 & 0 & 0 & 0 & 0 & \frac{1}{G_{xz}} \end{array}\right]$$

For the compliance matrix, S, the following applies: $\frac{\nu_{xy}}{E_{x}}=\frac{\nu_{yx}}{E_{y}}$, $\frac{\nu_{yz}}{E_{y}}=\frac{\nu_{zy}}{E_{z}}$, and $\frac{\nu_{xz}}{E_{x}}=\frac{\nu_{zx}}{E_{z}}$. This means that for the nine unknowns in the stiffness matrix, only six load cases needs to be applied.
